# SMG-6 mRNA cleavage stalls ribosomes near premature stop codons *in vivo*

**DOI:** 10.1093/nar/gkac681

**Published:** 2022-08-11

**Authors:** John H Kim, Matthew S Modena, Enisha Sehgal, Annie Courney, Celine W Neudorf, Joshua A Arribere

**Affiliations:** Department of MCD Biology, UC Santa Cruz, California, USA; Department of MCD Biology, UC Santa Cruz, California, USA; Department of MCD Biology, UC Santa Cruz, California, USA; Department of MCD Biology, UC Santa Cruz, California, USA; Department of Biomolecular Engineering, UC Santa Cruz, California, USA; Department of MCD Biology, UC Santa Cruz, California, USA

## Abstract

Nonsense-mediated mRNA decay (NMD) protects cells from the toxic and potentially dominant effects of truncated proteins. Targeting of mRNAs with early stop codons is mediated by the ribosome and spatiotemporally aligned with translation termination. Previously we identified a novel NMD intermediate: ribosomes stalled on cleaved stop codons, raising the possibility that NMD begins even prior to ribosome removal from the stop codon. Here we show that this intermediate is the result of mRNA cleavage by the endonuclease SMG-6. Our work supports a model in which ribosomes stall secondary to SMG-6 mRNA cleavage in *Caenorhabditis elegans* and humans, i.e. that the novel NMD intermediate occurs after a prior ribosome elicits NMD. Our genetic analysis of *C. elegans*’ SMG-6 supports a central role for SMG-6 in metazoan NMD, and provides a context for evaluating its function in other metazoans.

## INTRODUCTION

Nonsense-mediated decay (NMD) is a translational surveillance pathway where premature stop codon (PTC) containing mRNAs are recognized and degraded (reviewed in (1)). Targeting and destruction of PTC-containing mRNAs are critical for human health as PTC mutations are widespread among disease-causing lesions in humans (2). There is little consensus in the NMD field on the mechanism of NMD within experimental systems, let alone across organisms. For example, in mammals the nuclease SMG6 is reported to be nonessential for NMD by some groups (3–5), yet essential for NMD by others (6–9). Prevailing models of NMD target degradation suggest that SMG6 is only required for some events, with exonucleolytic degradation acting on others (1). Experimental study of NMD is confounded by the fact that key aspects of NMD are not recapitulated in lysates and that mutants of NMD factors are lethal in some experimental systems (*Drosophila*, mammals). Furthermore, the mRNA fragments made during NMD are rapidly cleared by downstream decay *in vivo* and are thus challenging to study.

Two important and conserved effectors of downstream decay are *skih-2* and *pelo-1* (10). *skih-2* is the catalytic subunit of the RNA helicase SKI that associates with the 3′ > 5′ exosome and ribosomes and removes ribosomes when they translate to the 3′ end of mRNA fragments. *pelo-1* forms a complex with HBS-1 and rescues ribosomes stalled on the 3′ end of mRNA fragments. *skih-2* and *pelo-1* are commonly known for their roles in the Nonstop decay and No-Go decay pathways (11–13). In both *C. elegans* and *Drosophila*, NMD intermediates are rapidly cleared by SKI and PELO (10,14), and loss of these factors enables the study of NMD intermediates.

By knocking out downstream decay, we previously found that ribosomes stall on cleaved stop codons on NMD targets in *Caenorhabditis elegans* (10). Surprisingly, we found that the majority of detectable ribosomal stalls were on NMD targets, suggesting that the aftermath of mRNA fragments during NMD represent a substantial burden on the cell. Our work echoed reports of NMD intermediates in mammals and *Drosophila* (14,15), and also importantly noted that RNA cleavages during NMD are directly over the stop codon and are ribosome-bound. Ribosomes arrested on cleaved stop codons raise questions about the relationship of translation termination to mRNA decay during NMD. Does NMD begin prior to ribosome removal from the mRNA? How do ribosomes stall on PTCs? What nuclease generates this intermediate? Armed with the ability to capture this NMD intermediate, we set out to answer these questions.

Here, we report that RNA cleavage during NMD is carried out by the RNA endonuclease SMG-6, and that this factor is required to stall ribosomes on mRNAs cleaved near their stop codons. After considering two models for their generation, we present evidence that stop codon cleavages and ribosome stalling arise as a secondary consequence of SMG-6 cleavages. We also demonstrate that cleavage by SMG-6 is an essential part of NMD in *C. elegans*, and present data that provide insight into the interpretation of the results of SMG-6 knockdown experiments.

## MATERIALS AND METHODS

### 3′ RACE

Total RNA was extracted from ground animals with trizol, resuspended in TE 7.4, and quantified using the Qubit HS RNA kit. No poly(A) selection nor ribosome subtraction was performed. 1ug of RNA was used as input for 3′ RACE library preparation. For ‘3′ OH + 3′ P 3′ RACE’, total RNA was first treated with T4 PNK (NEB) to remove 3′ phosphates. The T4 PNK reaction was cleaned up with phenol/chloroform extraction followed by RNA precipitation, and then used in ligation. For ‘3′ OH 3′ RACE’ libraries, total RNA was directly used in ligation. Volumes and conditions were per similar, published protocols (16). Preadenylated adaptor (AF-JA-34: /5rApp/NNNNNNAGATCGGAAGAGCACACGTCT/3ddC/) was ligated to RNA 3′ ends using T4 RNA Ligase 1 (NEB). Unligated adaptor was cleaned up using sequential 5′ deadenylase (NEB) and RecJ treatment (NEB). Ligated RNA samples were run on a urea 15% polyacrylamide gel and the ligated species was excised from the gel, eluted, and precipitated. Reverse transcription was performed using AF-JA-126 (/5Phos/AGATCGGAAGAGCGTCGTGT/iSp18/CACTCA/iSp18/GTGACTGGAGTTCAGACGTGTGCTCTTCCGATCT) as the primer and Superscript II RT (Thermo Fisher). cDNA was size-selected on a urea 10% polyacrylamide gel purified, and then circularized with circligase (Lucigen). PCR was performed to add illumina adaptors and barcodes for sequencing.

### Computational analyses

Reads were trimmed using cutadapt v2.8 (17). 3′ RACE and Ribo-seq libraries contained an N6 or N8 UMI on the 3′ adaptor (AF-JA-34), and PCR duplicates were collapsed using custom scripts. Reads were mapped to the *C. elegans* genome (Ensembl, release 100) including annotated splice junctions using STAR v2.7.3a (18) allowing for zero mismatches. For *unc-54(PTC)* experiments, reads were mapped to a custom genome containing the introduced mutations at the *unc-54* locus. This was done by masking the bases of the *unc-54* locus and creating a separate chromosome and annotation file for the *unc-54* locus. All downstream analyses were restricted to uniquely mapping reads. Normalized Read Density for metagene analyses was computed as previously described (19). Briefly, reads were normalized to control for differences in expression and the length of coding sequences by dividing by length-normalized read counts per CDS. Analyses were performed in python3 and plotted using PyX v0.15 using custom scripts.

The analysis of intermediate footprint sizes (Figure 5, Supplementary Figures S6 and S7) was as follows: we first restricted to genes with at least five Ribo-seq reads. (We observed similar results with read cutoffs of 1, 5, 10 or 20 counts.) We then tallied the number of reads at each position relative to the stop codon, counting reads by their 5′ ends and keeping reads separate according to their length. This created a data structure containing (position,count) pairs of position relative to the stop codon and Ribo-seq read counts. For each position and read length, we averaged read counts across all genes, generating the top plot of ‘Average Footprint Density.’ To ascertain the statistical significance, we performed a permutation test. For each read length within each gene, we shuffled (position, count) pairs and computed the average footprint density of the shuffled set. The shuffling was repeated 30 times (similar results were observed with more permutations, though we kept 30 for the plots in this study as each permutation is time-intensive). The 30 shufflings were used to generate a z-score of the observed ‘Average Footprint Density’ from the top plot for each read length and position, and a p-value calculated from the *z*-score to generate the ‘Statistical Significance’ plot.

For the heatmap of gene-specific 15–18nt Ribo-seq densities (Figure 6A), we restricted analysis to genes with at least 50 reads with 3′ ends within a 100nt window centered on the stop codon. We also removed genes with multiple stop codons (due to alternative mRNA isoforms) so as to avoid misclassifying footprints downstream of a stop codon. For each gene, we computed a cumulative distribution of read 3′ ends within a 100nt window centered on the stop codon. Genes were ordered by the position at which 50% of all reads were at or upstream.

### Ribo-Seq

Frozen animal pellets were ground with frozen PLB (20 mM Tris pH8.0, 140 mM KCl, 1.5 mM MgCl_2_, 1% Triton, 100 ug/ml cycloheximide) and liquid nitrogen in a mortar and pestle. Ground powder was mixed with ice cold PLB and clarified via a 10’ spin at 10 000 rcf at 4°C. Ribosome/RNA was quantified with a nanodrop and OD_260_ units were used to calculate the amount of RNaseI to use (total OD_260_ × 0.3). RNA was treated for 30 min at room temperature with RNaseI (Ambion, 100 U/ul) and loaded onto a 10–50% sucrose gradient. Gradients were spun in an SW41 Ti rotor in an ultracentrifuge at 35 000 rpm for 4.5 h. Monosomes were collected on a fractionator and digested with proteinase K. Monosome RNA was cleaned up by acid phenol chloroform extraction, precipitated, and stored in TE 7.4. 2–3 ug of purified monosomal RNA was run on 15% polyacrylamide gel and size-selected for 15–18nt, 19–26nt or 28–30nt footprints. Gel-purified RNA was treated with T4 PNK (NEB) to remove 3′ phosphates. RNA was then extracted with phenol chloroform, precipitated, and resuspended in TE pH7.4. Preadenylated adaptor (/5rApp/NNNNNNNNAGATCGGAAGAGCACACGTCT/3ddC/) was ligated onto RNA 3′ ends with T4 RNA Ligase 2 truncated KQ (NEB). Adaptor-ligated RNA was run on a 10% polyacrylamide gel, size selected, and processed as per reverse transcriptase, circligase, and PCR in the 3′RACE protocol.

### *C. elegans* growth and harvesting

All C. *elegans* strains (Supplementary Table S1) were made in the N2 background (VC2010) (20). Animals were synchronized via an egg prep (using sodium hypochlorite treatment), grown on NGM plates containing OP50 at 16°C (21), and harvested at the L4/young adult stage. Animals were passed through a 5% sucrose cushion in N50 to remove *Escherichia coli* and snap frozen in liquid nitrogen. Unless otherwise indicated, animals were lysed by grinding in a mortar and pestle cooled in liquid nitrogen. Ground animals were stored as frozen powder at −70°C.

In order to quantify the Unc phenotype (Figure 2C, D), single animals were placed in a drop of M9 buffer. Animals were monitored for 1 min, and the number of thrashes was counted. One thrash was defined as a full oscillation of the swimming motion of the animal.

### CRISPR/Cas9 and strain construction

The CRISPR/Cas9 genome editing technique was conducted as previously reported in (22). Mutations were introduced into the genome using custom gRNAs and donor oligos. Double and triple mutants were constructed using standard *C. elegans* techniques and balancers. All strains were confirmed via PCR and sequencing.

### RNA-seq

Trizol was used to extract total RNA from animals for *smg-1*, *smg-6* and wild-type samples for each of two replicates. RNA-seq steps including ribosome subtraction, library preparation, and deep sequencing were performed by Genewiz. Endogenous NMD targets were defined with DESeq2 (23). Targets were defined using the adjusted *P*-value, which corrects for multiple hypothesis testing. We restricted target analysis to genes with at least 5 counts in each of the six samples.

### Immunoblots

Animals harvested at the L4 stage with EN50 (1mM EDTA, 50mM NaCl) and stored at −70°C were boiled in 1xSDS loading buffer (0.5M Tris–HCl, 100% glycerol, 20% SDS, 1 M DTT, 5% bromophenol blue). Samples were run on a 4–20% Mini-PROTEAN TGX Stain-Free Protein gel (Bio-Rad Laboratories, Inc.). Protein was transferred to a low background fluorescence PVDF membrane (Millipore). The membrane was blocked in 1% nonfat milk in 1× TBST. The anti-FLAG antibody (Sigma F1804) was used at a 1:1000 dilution to detect FLAG-tagged SMG-6 protein. Secondary antibody staining was performed with 1:15 000 LI-COR goat anti-mouse (LI-COR). Imaging was performed using a LI-COR Odyssey Imaging System (LI-COR) and quantification done in ImageJ.

## RESULTS

### A metal-dependent nuclease acts at stop codons

We set out to understand the nature of the nuclease that cleaves NMD targets by examining the chemistry of the resultant 3′ termini genome-wide. The chemistry of 3′ termini reports on the nuclease that made them: metal-independent nucleases usually leave a 2′–3′ cyclic phosphate (resolved to a 2′ P or 3′ P) or a 3′ P, while metal-dependent nucleases leave a 3′ OH (24). We made two distinct libraries in parallel using two distinct 3′ RACE protocols: one captures RNA fragments with a 3′ hydroxyl (3′ OH library), and the other captures RNA fragments with a 3′ hydroxyl or a 3′ phosphate (3′ OH + 3′ P library). The 3′ OH library was made by omitting T4 polynucleotide kinase (PNK) prior to 3′ ligation, while the 3′ OH + 3′ P library was made by treating the RNA with T4 PNK prior to 3′ ligation (Materials and Methods). Metal-dependent nucleases would leave a 3′ P and thus be captured in the 3′ OH + 3′ P (+PNK) library, whereas metal-independent nucleases would leave a 3′ OH and be captured in both libraries, 3′ OH + 3′ P (+PNK) and 3′ OH (-PNK). To capture stop codon cleavages independent of cellular efforts to purge these RNA fragments, we performed these analyses in a *skih-2 pelo-1* double mutant.

**Figure 1.**
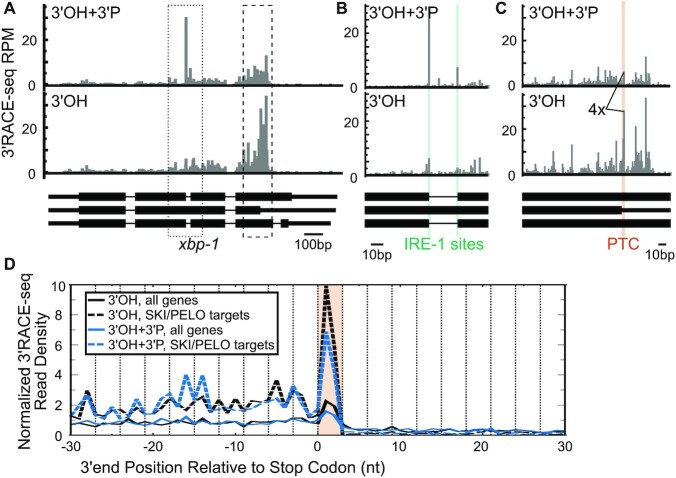
Cleavages at stop codons have a terminal 3′ OH (**A**) 3′ ends of fragments mapped to the *xbp-1* locus with PNK (3′ OH + 3′ P) and without PNK (3′ OH) phosphatase treatment prior to adapter ligation. Thin, intermediate, and thick black bars represent introns, untranslated regions (UTRs), and coding exons, respectively of annotated *xbp-1* isoforms. Scale bar in lower right. See text for description of isoforms. (**B**) Zoom in of IRE-1 cleavage sites in *xbp-1* (highlighted in green). (**C**) Zoom in of an *xbp-1* stop codon (red). (**D**) Metagene analysis of the 3′ ends of reads aligned by annotated stop codons in PNK-treated (blue) and untreated (black) libraries. Previously identified SKI/PELO targets (inc. many PTCs) shown as dashed lines. Y-axis is normalized read density, as per (19). Note that no RNase1 was used in any of the experiments shown.

To validate the specificity of the 3′ RACE protocols, we first focused on reads mapping to the endogenous *xbp-1* gene. Site-specific cleavage of *xbp-1* mRNA by the metal-independent endonuclease IRE-1 generates a 2′–3′ cyclic phosphate end (25). Consistent with this, we detected RNA fragments at the known IRE-1 cleavage site in *xbp-1* in our 3′ OH + 3′ P 3′ RACE library, and far fewer in our 3′ OH 3′ RACE library (Figure 1A, B). This result validates the specificity of our 3′ RACE protocols for different 3′ end chemistries.

We next turned our attention to cleavages at PTCs. *xbp-1* mRNAs cut at the IRE-1 site can be spliced back together to generate a full-length mRNA, yielding translation termination at a stop codon earlier than that of unspliced *xbp-1* mRNA. Previously we reported *smg*-dependent cleavages at the early stop codon of the spliced *xbp-1* isoform (middle isoform of Figure 1A) (10). In the 3′ OH 3′ RACE library we saw robust accumulation of cleavages at this stop codon, ∼4-fold more reads than these same positions had in the 3′ OH + 3′ P 3′ RACE library (Figure 1C). We also noticed changes at sites up- and down-stream of the stop codon. Such cleavages are clearly localized about the early stop codon, yet do not align exactly at the stop codon. We revisit the discussion of such instances later (see Figure 6, below).

Genome-wide, stop codons also showed an accumulation of 3′ OH-ends (Figure 1D), and this effect was greater for endogenous SKI/PELO targets, which include many known PTCs (10). It is worth noting that the 3′ RACE was performed on total RNA without exogenous RNase treatment or translation elongation inhibitors, ruling out RNase1- or cycloheximide-induced artifacts as a potential explanation for the stop codon cleavages. Taken together, our 3′ termini analysis demonstrates that cleavages at PTCs are generated by a metal-dependent endo- or exonuclease.

### The SMG-6 PIN domain is required for NMD

The above results are consistent with cleavage in and around stop codons by a metal-dependent nuclease. Prior work also identified a metal-dependent nuclease (SMG6) important for NMD in humans and *Drosophila* (26–30), and SMG6 has been shown to cut mRNAs near stop codons in human NMD (31,32). We thus hypothesized that *C. elegans* SMG-6 is responsible for the observed stop codon cleavages. Despite several studies, the existing literature in *Drosophila* and humans is ambiguous as to SMG6’s requirement in NMD (3–9). Given the tractability of *C. elegans* and its NMD system in particular, we first undertook an analysis of determinants of SMG-6 PIN domain function and its relationship to NMD.

**Figure 2.**
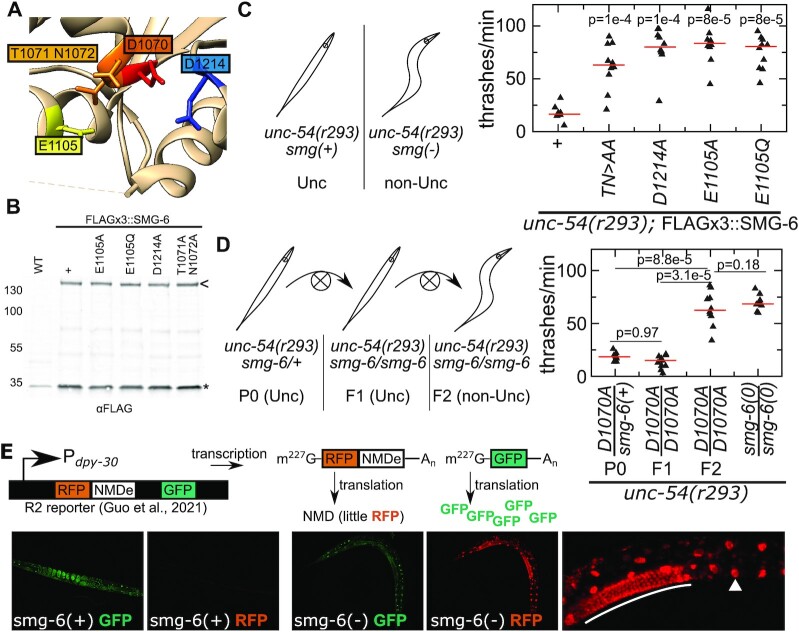
Mutations in conserved SMG-6 active site residues abolish NMD. (**A**) Structure of human PIN domain (2hww) (27). Amino acids are numbered according to homologous positions in *C. elegans*. (**B**) Immunoblot to examine expression of indicated FLAG-tagged SMG-6 alleles. MW size markers on left. SMG-6 is indicated with a carrot at right, and a non-specific band (loading control) indicated with an asterisk. (**C**) At left is a depiction of the *unc-54(r293)* assay, see text for details. At right is the results of ten individual animals, with the swimming speed of each animal represented by a single triangle. The median of all animals of that genotype is shown as a red bar. *P*-values from one-tailed Mann–Whitney *U* test comparing indicated strain to first strain (‘+’, wild type). (**D**) Left is a depiction of the generations from P0 (parental heterozygote), F1 (first generation *smg-6* homozygote), and F2 (second generation *smg-6* homozygote). At right is a graph as in (C), with *P*-values between strains indicated by the black bars. *smg-6(0)* is a published *smg-6* null allele, *r1217*. (**E**) The R2 (35) reporter is depicted (top), with NMDe (‘NMD element’) that elicits NMD on the RFP-encoding mRNA. Bottom shows microscopy of animals without a *smg-6* mutation (*smg-6(+)*) or with a *smg-6* mutation (in this case the *srf0841* allele, TN > AA). Far right is a zoom of the image at left in the *smg-6* mutant, with germline in white bar, and an intestinal nucleus indicated by white arrowhead.

We identified several highly conserved residues near the SMG-6 metal-binding active site (Figure 2A, Supplementary Figure S1), including three aspartate residues that are essential for cleavage by the human SMG6 PIN domain *in vitro* (29). Asp1070 sits in a conserved motif (DTN) at the center of the metal binding site. We also noticed that Glu1105 is highly conserved, suggesting it may be critical for *smg-6* function. So as to contextualize our results with prior work, we generated point mutations in some residues known to be important in *D. melanogaster* and humans. We also expanded on previous work to include the potentially important Glu1105 and the highly conserved Thr and Asn adjacent to Asp1070. We generated point mutations at each of these residues using CRISPR/Cas9. Many alleles were also FLAG-tagged, and immunoblot analysis confirmed that the mutations did not perturb SMG-6 protein expression (Figure 2B).

To assay NMD function, we crossed each mutation into an NMD phenotypic reporter strain, *unc-54(r293)* (33,34). *unc-54(r293)* is an allele of *unc-54* that encodes a functional UNC-54 myosin heavy chain protein required for animal movement, and its phenotype is uncoordinated because the *unc-54(r293)* mRNA is targeted by NMD. Animals with functional NMD are thus uncoordinated (Unc) and animals lacking NMD are not uncoordinated (non-Unc). A simple assay for Unc is to place the animals in a liquid and count the frequency with which they thrash. *unc-54(r293)* animals were non-Unc when crossed with each *smg-6* mutation, indicating de-repression of *unc-54(r293)* and loss of a functional NMD pathway (Figure 2C). Thus, each of these *smg-6* residues is required for NMD.

In the course of the above experiments, we noticed that all the active site mutations of *smg-6* exhibited maternal rescue. Maternal rescue is a phenomenon wherein homozygous mutant progeny fail to manifest a phenotype in the first generation. This can be due to cytoplasmic inheritance of functional wild-type protein or mRNA packaged from the mother into the developing egg. Subsequent generations exhibit the phenotype as the wild-type protein (SMG-6) is diluted out and lost. Maternal rescue has been documented before with prior *smg-6* mutants (33), and we extend those observations to include the PIN domain mutations described here (Figure 2D). The maternal rescue demonstrates that complete removal of functional SMG-6 protein is slow. If this is true in other organisms such as humans, it would complicate the interpretation of experiments using *smg-6* knockdown at the mRNA level (see Discussion).

We also tested whether there were tissue-specific effects of *smg-6*. UNC-54 is expressed in the body wall muscle, and a requirement for *smg-6* in other tissues could not be tested with the *unc-54(r293)* assay. Therefore, we turned to a recently published cell-specific NMD reporter (35), known as the R2 reporter. The R2 reporter expresses RFP and GFP from the *dpy-30* promoter broadly throughout the animal, with each coding sequence being processed into its own mRNA. Additionally, the RFP contains a 3′UTR that elicits NMD. Thus, animals with a functional NMD pathway will express GFP but not RFP, while animals with a nonfunctional NMD pathway will express both GFP and RFP. We validated this reporter by assaying mutants in known NMD factors: *smg-1(e1228)* (encoding the SMG-1 kinase), *smg-4(az152)* (encoding UPF3), and *smg-5(r860)* (encoding SMG-5) (33,36,37). In each case the mutant conferred de-repression of RFP throughout somatic and germline tissue of the organism (Supplementary Figure S2). A *smg-6* mutation also conferred RFP de-repression throughout the organism (Figure 2E). Thus we conclude that *smg-6* is required for NMD across tissues in *C. elegans*.

### SMG-6 PIN domain targets exhibit high overlap with SMG-1 targets

One prominent model in the NMD field is that SMG-6 cleavage is one option of several degradation pathways by which NMD represses its mRNA targets (1). We note this model is at odds with the original genetic characterization of *smg-6* and the other *smg* factors in *C. elegans*, as loss of function of any one factor is sufficient to break the NMD pathway as assayed by genetic suppression of NMD reporters such as *unc-54(r293)* (33). Our genetic analysis (Figure 2) showed that the PIN domain of SMG-6 is required for repression of both the *unc-54(r293)* and R2 reporters. To explore a requirement for the SMG-6 PIN domain in NMD more globally in *C. elegans*, we examined its role in repression of endogenous NMD targets via RNA-seq.

**Figure 3.**
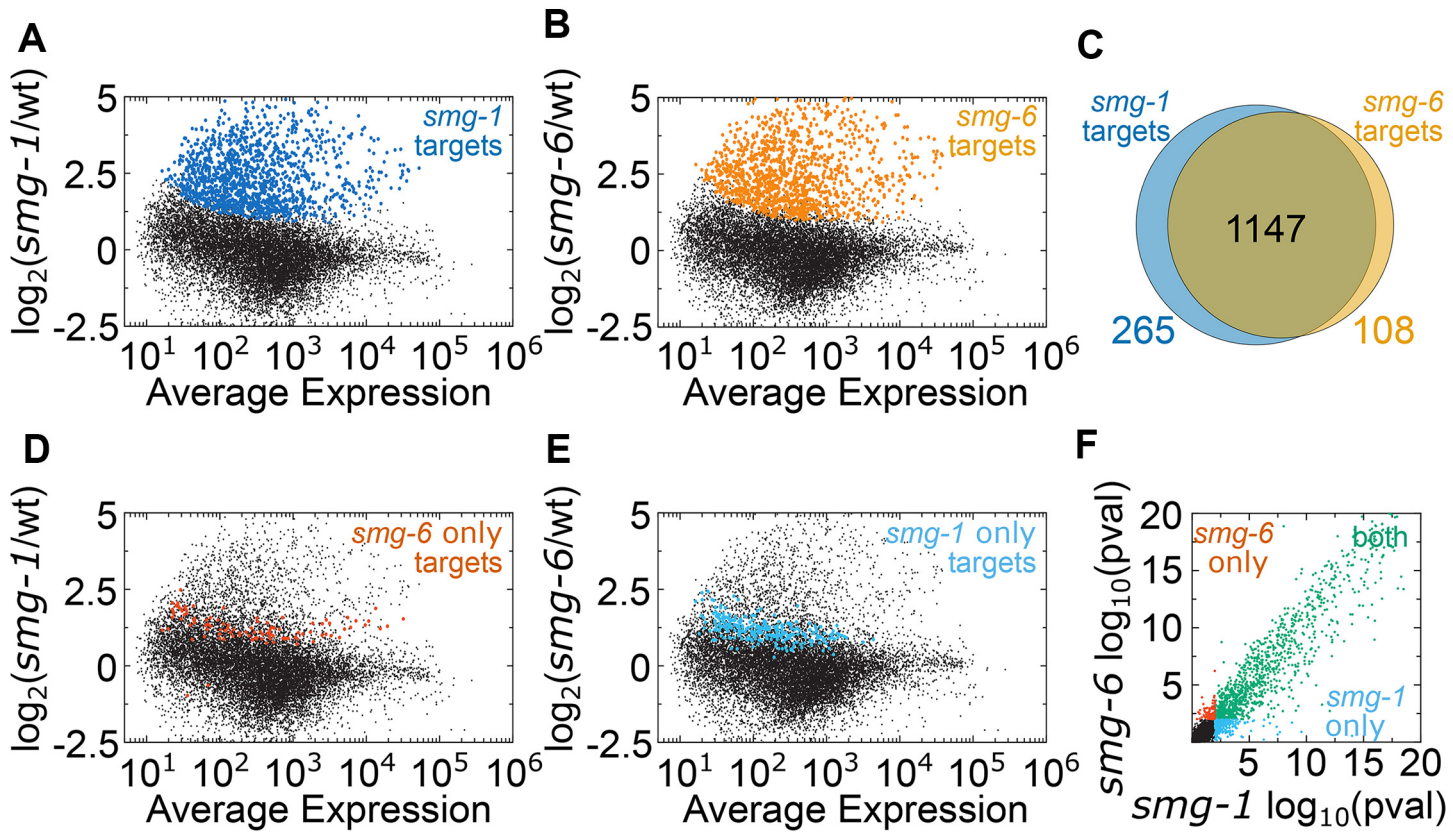
There is a high overlap between *smg-1* and *smg-6* targets. (**A**) Scatterplot depicting log2 Fold Change (y-axis) in a replicate of *smg-1* RNA-seq libraries compared to wild type libraries. X-axis is the average expression across *smg-1* and wild type libraries. Each dot represents a single gene, with its location determined by RNA-seq counts. Genes whose mRNAs increase at an adjusted *P*-value cutoff of 0.01 in a *smg-1* animal are highlighted in blue. (**B**) Same as in (A), but with a *smg-6(D1070A)* mutant. (**C**) Venn diagram of overlap between *smg-1* and *smg-6* targets. (**D**) Same graph as in (A), but with *smg-6* only targets (108) highlighted in red. Note all but two gene's mRNAs increase in the *smg-1* mutant. (**E**) Same graph as in (B), but with *smg-1* only targets (265) highlighted in light blue. Note all gene's mRNAs increase in the *smg-6* mutant. (**F**) Comparison of *P*-values for differential expression between *smg-6* and *smg-1* strains. Each dot is a gene, with its position on the x-axis and y-axis determined by the adjusted *P*-value from DESeq2. Genes are colored according to whether they were upregulated in *smg-6* (red), *smg-1* (light blue) or both (green) mutants.

NMD is known to be required for the repression of several endogenous mRNAs, providing a diverse array of NMD substrates by which we could test for a requirement for *smg-6* and its PIN domain. To identify endogenous NMD targets, we first performed RNA-seq in a *smg-1* mutant and examined upregulated mRNAs using DESeq2 (Figure 3A). SMG-1 is known to be required for NMD in *C. elegans* (33). Using DESeq2, we identified 1412 endogenous genes whose mRNAs were significantly upregulated at an adjusted p-value cutoff of 0.01 (Materials and Methods). This group of mRNAs overlapped with a previous list of NMD targets made by genome-wide microarrays (256 of 1412 genes, Supplementary Figure S3) (38). The somewhat modest overlap is expected given the difference in methodology (microarrays vs. RNA-seq) as well as statistical methodology (microarrays not being conducive to the dispersion-based approach of DESeq2). We note that here (as in other studies), mRNAs upregulated in a *smg-1* mutant will include both direct targets (i.e. NMD substrates) as well as indirect targets (downstream consequences of de-repression of NMD substrates). For the sake of simplicity, we refer to both as targets; the fact that some are indirect does not invalidate the approach.

We identified *smg-6* PIN targets by examining mRNA expression in the *smg-6(D1070A)* mutant. This approach identified 1255 *smg-6* targets at an adjusted *P*-value cutoff of 0.01 (Figure 3B). The 1255 genes exhibited a high degree of overlap with *smg-1* targets, with 1147 genes in common to both target lists (Figure 3C). We thus conclude that a majority of mRNAs regulated by *smg-1* are also regulated by *smg-6* and vice-versa.

There were 265 genes unique to the *smg-1* target list, and 108 genes unique to the *smg-6* target list. Inclusion of a gene on one target list and exclusion from the other could arise because: (A) the gene is uniquely regulated by one of *smg-1* or *smg-6* or (B) a failure to detect a statistically significant difference due to a relative increase in effect size in one set of libraries (e.g. due to deviations in fold change or dispersion). To discern between (A) and (B), we examined the *smg-6* targets in the *smg-1* mutant. Of the 108 *smg-6*-specific targets, 106 exhibited an increase in expression in the *smg-1* mutant, falling just below the cutoff and thus not making the *smg-1* target list (Figure 3D). On average, these genes’ mRNAs are indeed *smg-1*-regulated, but simply did not pass the threshold for statistical significance. The two *smg-6* targets that decreased expression in the *smg-1* data were *srw-85* and *frpr-13*. Further work will be required to determine whether these genes indeed represent *smg-6*-specific and *smg-1*-independent targets, or whether they represent false positive *smg-6* targets. All 265 *smg-1* targets that did not make the *smg-6* target list exhibited an increase in expression in the *smg-6* mutant but fell below the statistical cutoff (Figure 3E).

We also looked for the existence of *smg-1*-specific or *smg-6*-specific targets by comparing the statistical significance of each identified target. If a gene's mRNAs depended specifically on *smg-1* or *smg-6*, that gene would exhibit a more significant statistical change in either of *smg-1* or *smg-6* relative to the other. If instead a gene's mRNAs depended similarly on both factors, it would exhibit a similar statistical significance in both *smg-1* and *smg-6*. We observed that most genes exhibited a similar statistical significance for differential expression in *smg-1* and *smg-6* (Figure 3F). The targets specific to one factor tended to have a lower statistical significance than targets of both factors, consistent with the interpretation that factor-specific targets just passed statistical significance in one mutant and just fell below statistical significance in the other. Taken together, our analyses of the *smg-1*-specific and *smg-6*-specific targets supports a model that such targets are in fact regulated by both *smg-1* and *smg-6*, but fall at the limit of statistical cutoffs and are thus binned as *smg-1*-specific or *smg-6*-specific.

Given the high degree of overlap for *smg-1* and *smg-6* target lists (Figure 3) and the requirement for the PIN domain residues for phenotypic repression two reporters (Figure 2), we conclude that the SMG-6 PIN domain is generally required for NMD across mRNAs and tissues of *C. elegans*. We cannot rule out that there is some condition where SMG-6 and its PIN domain is not required for NMD, and we note that such effects in lowly expressed genes would have evaded the above approach due to limitations of sequencing depth. Under the conditions examined here and for the genes detectably expressed, we saw little support for the model of *smg-6*-independent NMD targets in *C. elegans*.

### Cleavage by the SMG-6 PIN domain is required for NMD stop codon cleavages

Having established a requirement for SMG-6 in NMD, we set out to determine if SMG-6 was involved in ribosomal stalling over stop codons. We performed ribosome footprint profiling (Ribo-seq) (19), with some modifications of the original Ribo-seq technique in order to interrogate ribosomes in different states, namely ∼28–30nt footprints (ribosomes pre-translocation or peptide bond formation), ∼21nt footprints (ribosomes with an empty A-site), and 15–18nt footprints (ribosomes on truncated mRNA fragments, where the 3′ end represents the A-site) (10,39–46).

**Figure 4.**
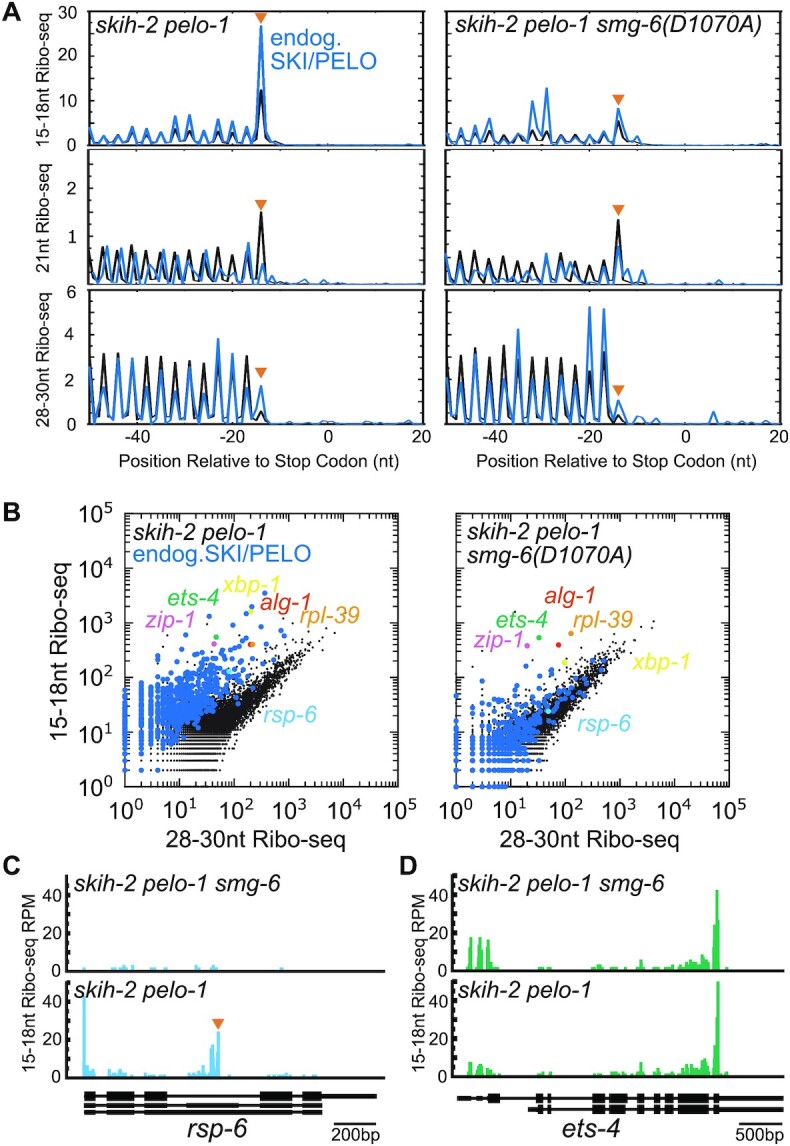
The PIN domain of SMG-6 is required for ribosome-associated cleavages at stop codons. (**A**) Meta-gene analysis of Ribo-seq reads captured in a *skih-2 pelo-1* double mutant (left) and a *skih-2 pelo-1 smg-6(D1070A)* triple mutant (right). The y-axis is in Normalized Read Density (as in Figure 1D). The blue line represents previously identified endogenous SKI/PELO targets (10). The orange arrowhead notes the expected location of a ribosome with a stop codon in its A-site. (**B**) Comparison of 15–18nt Ribo-seq read counts to 28–30nt Ribo-seq read counts in *skih-2 pelo-1* and *skih-2 pelo-1 smg-6(D1070A)*. Blue dots are the previously identified endogenous SKI/PELO targets. Genes of interest are colored; see text for further details. (**C**) Read density in the two libraries at the gene *rsp-6*, an endogenous SKI/PELO and NMD target. Scale in bottom right. (**D**) Same as (C), but at *ets-4*, an endogenous SKI/PELO but not a NMD target.

We crossed an active site point mutation of *smg-6(D1070A)* into the *skih-2 pelo-1* strain and performed Ribo-seq. In the absence of active *smg-6*, stop codon cleavages were reduced genome-wide (orange triangle, Figure 4A). The decrease of stop codon cleavages was even more striking (3.3-fold decreased) for a group of previously annotated SKI/PELO targets which include several known PTCs (10). While the 15–18nt Ribo-seq reads were reduced at stop codons in the *smg-6* mutant, they were not completely lost, consistent with the idea that some genes stop codon cleavages independent of *smg-6* and NMD (e.g.*ets-4*, and below).

In addition to the reduction in stop codon cleavages in the *smg-6* strain, we noticed that 21nt Ribo-seq reads at the stop codon for SKI/PELO targets did not differ with and without *smg-6* (Figure 4A). 21nt footprints over stop codons can indicate inefficient termination (39,44). This result is consistent with work in human cells, where ribosomal stalling at PTCs was also not detected (47).

To determine whether SMG-6 is required for cleavages on a gene-by-gene basis, we examined read counts and distributions for individual transcripts. Comparing 15–18nt relative to 28–30nt Ribo-seq counts between *skih-2 pelo-1* and *skih-2 pelo-1 smg-6*, we observed that a majority of SKI/PELO targets exhibited a reduction of 15–18nt Ribo-seq reads in the *smg-6* mutant down to a level comparable to that of all genes (Figure 4B). Examples of such genes include known NMD targets in *C. elegans* (*rsp-6*, *rpl-12*, *rsp-7*, Figure 4C and Supplementary Figure S4). Both *rsp-6* and *rsp-7* produce isoforms with an alternative internal exon. The internal exon contains a PTC, making that isoform an NMD target (48). *rpl-12* is known to produce an alternative 5′ splice site that again leads to translation termination at a PTC and is an NMD target (49). Thus SMG-6 is required for cleavages on endogenous NMD targets in *C. elegans*.

15–18nt Ribo-seq reads persisted for several genes in the *smg-6* strain. For example, *xbp-1* has two populations of 15–18nt Ribo-seq reads, one of which arises from the action of the endonuclease IRE-1 and is *smg-6*-independent, and the other of which arises at an early stop codon and is *smg-6*-dependent. Other genes exhibited 15–18nt Ribo-seq reads at their stop codon independent of *smg-6*, including *zip-1*, *ets-4*, *alg-1*, and *rpl-39* (Figure 4B, D, Supplementary Figure S5). The 15–18nt Ribo-seq reads for these four genes were also *smg-1*-independent, suggesting that the reads were not produced by NMD. Consistent with this, *ets-4* was previously identified as the target of a nuclease (REGE-1, regnase) in *C. elegans* (50). We conclude that *ets-4* and the remaining *smg-6*-independent genes (*zip-1*, *alg-1* and *rpl-39*) are degraded through mechanisms genetically distinct from *smg-6* and NMD.

### A model to explain 15-18nt Ribo-seq reads at and around PTCs

Our work thus far supported the idea that SMG-6 cuts the mRNA, stalling ribosomes and giving rise to 15–18nt Ribo-seq reads at and around the PTC. We set out to understand the events immediately preceding ribosomal stalls at and around stop codons by considering two possible models for their generation.

**Figure 5.**
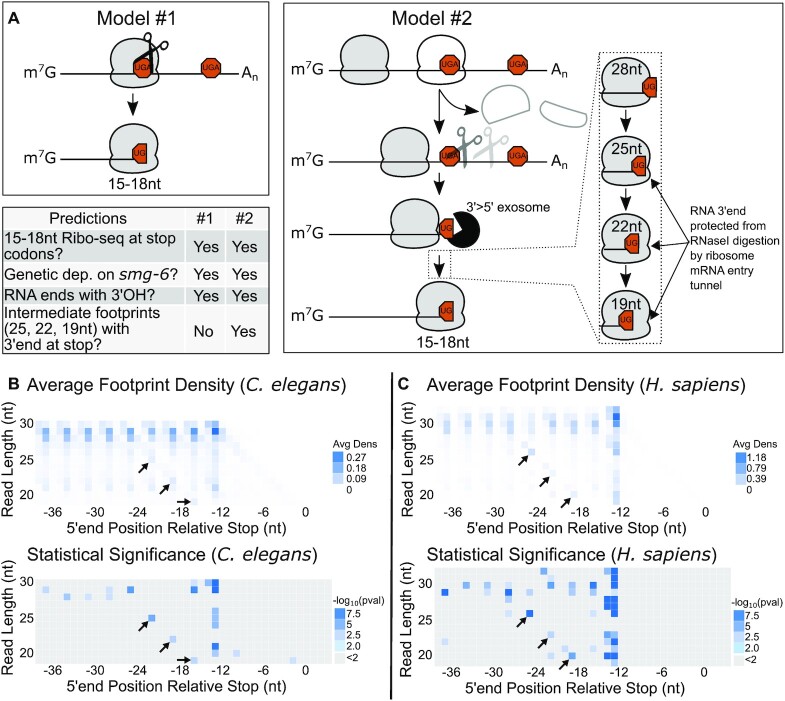
Ribosome footprint profiling detects rare, intermediate-sized ribosome footprints with cleavages in the stop codon. (**A**) Two models for the enrichment of ribosome protected short footprints at stop codons. For model two, dotted lines indicate intermediate steps between exosome and final arrest of ribosomes as 15–18nt ribosome footprint species. Predictions of both models are indicated in the bottom left table. (**B**) and (**C**) Heatmap showing density of 5′ ends across a range of Ribo-seq footprint sizes relative to stop codons in *C. elegans* (B) (44) and humans (C) (51). Black arrows indicate the expected range of footprints for ribosomes translating to the end of a cleaved message based on the distribution of 15–18nt Ribo-seq cleavage sites in *C. elegans*. Statistical significance ascertained by permutation testing, see text for further details.

One model to explain stop codon cleavages is that they are the direct result of SMG-6 cleavage, e.g. via cleavage in the ribosomal A-site (Figure 5A). Alternatively, stop codon cleavages may arise due to SMG-6 cleavage downstream near a stop codon, followed by 3′ > 5′ exonucleolytic digestion, and then stalling by another, trailing ribosome on the end of what is now a stop codon-less (i.e. nonstop) mRNA fragment. As the 3′ > 5′ exosome is a metal-dependent nuclease, both models would predict 3′ OH ends (Figure 1), *skih-2* and *pelo-1*-dependence of ribosome stalling (10), and a genetic dependence of stop codon cleavages on *smg-6* (Figure 4).

A key difference in the second model is that it predicts a series of intermediate species as the trailing ribosome elongates to the RNA 3′ end (Figure 5A). A full ribosome footprint is ∼28nts, with ∼10nts between the A-site and the mRNA entry tunnel. As the ribosome translates to the edge of an RNA fragment, the mRNA entry tunnel would be expected to protect the 3′ end by sterically occluding RNase1 from the RNA 3′ end. The effect would generate three intermediate sizes between full-length 28nt footprints and stalled 15–18nt footprints. The intermediate footprints (25nts, 22nts, 19nts) would have the same 3′ end, but be three nucleotides shorter at their 5′ end due to less of the mRNA being protected during RNase1 digestion. We thus set out to determine whether intermediate footprints exist at stop codons as means to differentiate between the first and second model.

Because the intermediate footprint sizes were selected out of our libraries, we first examined published *C. elegans* Ribo-seq that includes footprints from 21–30nts (44). We observed a signal of intermediate footprint sizes above what would be expected by random chance (Figure 5B). This result supports the second model, that 15–18nt footprints at stop codons arise due to translation to the 3′ end of a cleaved mRNA. We attempted to replicate the phenomena, and while one of two biological replicates showed intermediate-sized ribosome footprints, their abundance was not statistically significant (Supplementary Figure S6). We attribute this to the inherent difficulty in trapping short-lived and rare intermediates of an mRNA undergoing decay. The existence of such a footprint series under any condition is consistent with the idea that translation to the 3′ end of cleaved mRNAs generates 15–18nt Ribo-seq patterns, i.e. the second model of Figure 5A.

We also analyzed Ribo-seq datasets from human cells to determine if the phenomenon was conserved. We observed intermediate footprints three nucleotides upstream of the sites found in *C. elegans* (Figure 5C) (51). The slight shift in the position could reflect variability between species in nucleolytic cleavage at stop codons and/or variability due to the slightly larger footprint protected by mammalian ribosomes. As in *C. elegans*, the intermediate footprint sizes were not observed in all human datasets, which we again attribute to their rare and short-lived nature.

During our analysis of the intermediate footprint sizes, we noticed a more pronounced effect for TAA-ending genes compared to either of TGA- or TAG-ending genes in *C. elegans* and humans (Supplementary Figure S7). We are unsure as to the reason for the difference. Differences could arise due to the higher average expression of TAA-ending genes, providing more signal and greater statistical power. There may also be stop codon-specific effects, though we note that our prior work recovered more cleavages at TGA-ending codons relative to TAA or TAG codons (10).

Taken together, our data are consistent with the second model (translation to the end) occurring in both *C. elegans* and humans.

### Ribosomal stalls at cleavages in the 3′ UTR

**Figure 6.**
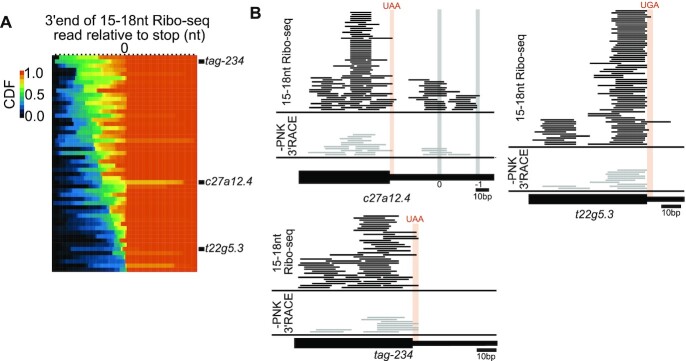
Cleavages occur in and around stop codons and exhibit gene-specific positional differences. (**A**) Heat map of gene-specific cumulative distribution functions (CDFs) of 15–18nt Ribo-seq reads in a 100 nt window centered around stop codons. Reads were tallied at their 3′ ends. Black ticks at top of graph indicate 3 nts. (**B**) Three genes chosen to illustrate different patterns of reads near the stop codon. Thick and thin black bars indicate coding sequence and 3′ UTR, respectively. Stop codons are highlighted in orange. For *t22g5.3*, note that the most prevalent 3′ ends are just upstream of the stop codon. For *c27a12.4*, position of stop codons in 3′UTR highlighted in grey for zero- and -1-frame.

Our metagene analysis (Figure 4A) demonstrated that ribosomal stalling at stop codons on cleaved mRNAs is a major downstream product of SMG-6 cleavage during NMD. Given that some ribosome footprints arise from translation to an already cleaved end (Figure 5), we wanted to examine the Ribo-seq data on a gene-by-gene basis to see if there were informative deviations from the metagene average. For at least one gene (*xbp-1*), we knew that cleavages occurred at the stop codon, as well as at positions up- and downstream (Figure 1A–C). Consistent with the metagene, we observed that a majority of genes exhibited a sharp increase in the number of 15–18nt Ribo-seq reads with 3′ ends in the nucleotides of the stop codon (Figure 6A), a result that is consistent with both models one and two of Figure 5A. We also noticed that many genes had additional density upstream of the stop codon, and in fewer cases, downstream as well (Figure 6A, B). Importantly, gene-specific 15–18nt Ribo-seq patterns were reproducible across biological replicates, consistent with the idea that they represent gene-specific patterns rather than noise (Supplementary Figures S8 and S9). The 15–18nt Ribo-seq reads upstream of stop codons are consistent with secondary decay processes (i.e. 3′ >5′ degradation) stalling trailing ribosomes. In some cases (e.g.*t22g5.3*), we observed a sharp accumulation of 3′ ends upstream of the stop codon. We would not expect such 3′ ends under the first model of Figure 5A. However, such 3′ ends could be attributable to preferred sites of SMG-6 cleavage outside of an elongating ribosome and/or sites of stalling by 3′ > 5′ exosome, followed by stalling of the trailing ribosome (as predicted from model two).

In a few cases (e.g.*c27a12.4* Figure 5B) we observed 15–18nt Ribo-seq reads downstream of the stop codon. We observed reads at these same positions in the 3′ RACE data, arguing against such reads being artifacts of the Ribo-seq protocol. We did not detect a frame bias for 3′ UTR-mapping Ribo-seq reads genome-wide. This result is consistent with two models: (A) 3′ UTR-mapping Ribo-seq reads represent ribosomes not actively translating at the time of their capture (i.e. post-termination, pre-recycled ribosomes) or (B) the mechanisms underlying bypass of a stop codon differ from gene-to-gene (i.e. ribosomes readthrough a stop codon of one gene by a –1 frameshift, but readthrough another stop codon in a different gene by readthrough in the zero frame). The small number of reads per gene make it difficult to discern between these models, but we favor the second model based on data later obtained at the *unc-54* locus (see below).

### Termination site- and sequence-dependent differences in NMD target cleavage

**Figure 7.**
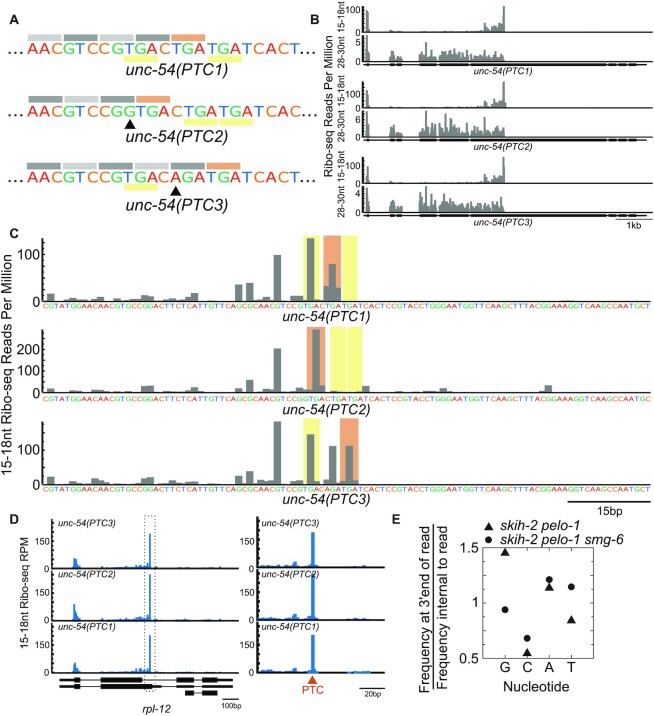
Similarities and differences at cleavage sites across three highly similar NMD reporters. (**A**) Schematic of the three *unc-54* reporters PTC1, PTC2 and PTC3 with decoy stop codons (underlined in yellow). Grey bars indicate the translation frame. Red bar denotes the in-frame stop codon. Arrows indicate a G insertion or T > A mutation in PTC2 and PTC3 (respectively) relative to PTC1 reporter. (**B**) Ribo-seq reads for each of the *unc-54* reporters in a *skih-2 pelo-1* background, with 15–18nt reads on top, and 28–30nt reads below. Reads were mapped by their 5′ end. (**C**) Zoom in of translation termination site and surrounding sequence from (B), displaying 15–18nt Ribo-seq read counts at the last nucleotide of the 3′ end of the read. Coloring as per (A); red indicates site of translation termination, and yellow indicates decoy stop codons. Sequence displayed below. (**D**) The *rpl-12* locus (an endogenous NMD target) in the various *unc-54* reporter 15–18nt Ribo-seq libraries. (**E**) Prevalence of single nucleotides at the 3′ ends of reads about stop codons in *skih-2 pelo-1* and *skih-2 pelo-1 smg-6(D1070A)* 15–18nt Ribo-seq libraries (from Figure 3) for reads within 10nts of a stop codon (Materials and Methods).

The above gene-specific differences in Ribo-seq reads could arise from differences in the sequence of the genes and/or from spatiotemporal differences in each gene's expression and regulation. To better understand the determinants of cleavage, we generated a series of three reporters (Figure 7A). The three reporters were based on the sequence of the premature stop codon-containing *unc-54(r308)* allele, with additional stop codons near the expected stop codon. The reporters were made via CRISPR/Cas9 edits of the endogenous *unc-54* locus. The reporters (*unc-54(PTC1)*, *unc-54(PTC2)* and *unc-54(PTC3)*) encode identical ∼6200nt mRNAs except 1–2 nucleotides near the site of termination. Importantly, the mutations change the precise site of termination as well as the last few codons and amino acids.

We crossed each of the reporters into the *skih-2 pelo-1* background and performed 15–18nt and 28–30nt Ribo-seq. As expected, all three samples exhibited a high correlation of Ribo-seq read counts and distributions (Supplementary Figure S10). We observed similar 15–18nt Ribo-seq counts at the *unc-54* locus (Supplementary Figure S10B), and in the vicinity of the PTCs (Figure 7B). Zooming in on the PTCs, we observed highly similar distributions upstream of the PTC, but differences at the precise site of termination (Figure 7C). As the site of termination was moved progressively downstream, we observed a shift in the distribution of captured footprints as well, with the majority of footprints occurring at or upstream of the termination codon. Such deviations in 3′ ends were not observed at other endogenous NMD-targeted loci (*rpl-12*, Figure 7D).

We note that 3′ ends in the vicinity of the PTCs tended to occur at a guanosine (Figure 7C). This was also true genome-wide and was *smg*-dependent (Figure 7E). While guanosines are known to stall some exoribonucleases (52), such stalling generally occurs at runs of guanosine (16 or more), and we observed fewer reads at GG dinucleotides than single guanosines upstream of the stall (e.g.*unc-54(PTC2)*). Interestingly, recent work has shown that some TUTases prefer 3′ Gs (53,54). The short nature of our reads (15–18nt) preclude robust detection of untemplated uridylated RNA species; during the protocol we perform a strict size selection that removes longer RNA species. Still, the link between secondary decay processes (3′ G bias) and the position of the short footprints again favors a model in which many truncated footprints are due to interactions with secondary decay processes rather than the primary products of cleavage.

In the *unc-54(PTC2)* strain we observed a large number of 15–18nt Ribo-seq reads downstream of the stop codon (Figure 7C, Supplementary Figure S11), ∼4% of all 15–18nt Ribo-seq reads at the *unc-54* locus in this strain. The *unc-54(PTC2)* reporter has a stop codon (TGAC) that confers high levels of readthrough across eukarya (55). Indeed, over 90% of the 15–18nt Ribo-seq reads downstream of the PTC were in-frame, meaning that these ribosomes were actively elongating at the time of their capture. Such actively elongating ribosomes downstream of a stop codon could arise due to ribosomes that read through the PTC2 stop codon or ribosomes that terminate and then reinitiate (there are two in-frame ATG codons downstream of PTC2, green bars in Supplementary Figure S11). Either model would be expected to generate in-frame reads downstream of PTC2.

Interestingly, we did not observe reads at the next in-frame stop codon (∼100nts downstream of *unc-54(PTC2)*). If the ribosome directed cleavages via its A-site (as in model one of Figure 5A), we would expect 15–18nt Ribo-seq reads at the next in-frame stop codon as ribosomes that fail to terminate at PTC2 elongate to the next in-frame stop codon and then prematurely terminate. The absence of reads at the next in-frame stop codon argues against model one of Figure 5A. If instead model two of Figure 5A is correct and a ribosome elicits cleavage by SMG-6 downstream of *unc-54(PTC2)*, we would expect to capture such cleavages only when the trailing ribosome bypasses the *unc-54(PTC2)* stop codon. Under model two we would expect 15–18nt Ribo-seq reads to become less abundant further downstream of PTC2, as the chances of a ribosome encountering a 3′ end generated by SMG-6 and the exosome increase as the ribosome elongates downstream. Thus model two predicts an absence of reads at the next in-frame stop codon downstream of PTC2, as appears to be the case in our data.

The *unc-54(PTC2)* 15–18nt Ribo-seq read pattern is reminiscent of similar patterns observed on some endogenous NMD targets (*c27a12.4*, Figure 6B, *k11h12.3*, *mig-38*, Supplementary Figure S8). Our analysis of *unc-54* footprints is again consistent with the second model, that our short captured footprints are generated by a ribosome translating to a site that has been already cleaved by endogenous nucleases.

## DISCUSSION

Here, we describe our observations surrounding RNA 3′ ends, ribosomal stalling on NMD targets, and the relationship of these species to the endonuclease SMG-6. Our analyses led to the conclusion that some ribosome footprints are the result of stalling on the cleavage products left by SMG-6 and 3′ > 5′ decay, as opposed to a model in which all ribosome footprints on cleavage products result from ribosomes stalled at a SMG-6 cleavage site. Here we discuss our observations in light of the literature around SMG-6, and highlight areas for future work.

The literature is unclear on the requirement of human SMG6 in NMD, though the maternal rescue of *C. elegans*’ *smg-6* suggests a potential explanation. Upon hSMG6 knockdown in human cells, early studies observed mild effects on NMD, and hSMG6/7 or hSMG5/6 double knockdowns were required to see robust effects (3–5). This led to the notion that hSMG6 and hSMG5/7 function in distinct yet functionally redundant pathways. We note that this is at odds with the early *C. elegans* NMD literature showing a requirement for each of *smg-5*/*6*/*7* in NMD (33), as well as more recent studies arguing hSMG6 is required for human NMD (6–9). Maternal rescue of *smg-6* mutants in *C. elegans* shows that functional depletion of SMG-6 is slow, as *smg-6* function persists for a generation after the gene is mutated. If there is a similar delay in functional SMG-6 loss in human cells, it would be difficult to observe a requirement for *smg-6* in NMD using knockdown techniques such as RNA interference (RNAi). Consistent with this logic, the earlier studies that observed mild effects of hSMG6 (3–5) employed RNA interference (RNAi) and shorter transfection times (3–5 days) than later studies (7–9) which used CRISPR, longer transfection times (8–10 days), and observed effects of hSMG6 loss. A single study bucks this trend but is informative: (6) used RNAi and a short transfection time (3 days), but also implemented technical improvements on the RNAi technique (multiple siRNAs per gene, off-target correction) that would be expected to enhance hSMG6 knockdown. We cannot rule out that variability in conclusions from these studies arises due to differences in the NMD pathway between cell types and/or NMD targets examined. Given our work in *C. elegans* and our reading of the published human literature, we urge caution and care in the interpretation of knockdown experiments of hSMG6.

Our analysis of *smg-6* targets in *C. elegans* supports the idea that nearly all mRNAs that depend on *smg-6* for expression also depend on *smg-1*. We also note that null alleles of any of *smg-1*, *smg-2*, *smg-3*, *smg-4*, *smg-5* or *smg-6* yield similar de-repression of NMD reporters (33). We hypothesize that in *C. elegans* there is a single degradative NMD pathway that requires the concerted actions of all SMG proteins. Somewhere along the pathway is RNA cleavage by the SMG-6 PIN domain, as supported by our genetic analysis (Figures 2 and 3). Future work will be required to test this single pathway model. Existing literature in humans on the relative importance of the SMG proteins is mixed (see preceding paragraph), with recent work concluding that hSMG5/7 act to enhance hSMG6 cleavage (56) while another group concluded a hierarchical importance of factors (hSMG5 > hSMG6 > hSMG7) (57).

Our analyses of *in vivo* ribosome footprints are consistent with a model where a ribosome triggers NMD and recruits SMG-6 to cleave the mRNA near the PTC (the second model of Figure 5A). Exonucleolytic digestion by 3′ > 5′ exonucleases accompanied by ongoing translation stalls ribosomes on 3′ ends, and these ribosomes are then cleared by SKI and PELO. This model is based on observations in both *C. elegans* and humans and thus depicts conserved dynamics of translation and mRNA decay during metazoan NMD. As the 3′ ends of the intermediate footprint (25nts, 22nts, 19nts) we observe directly overlap the stop codon, they represent an earlier intermediate on the NMD pathway than those reported previously (15).

This model clarifies the events immediately following cleavage of the mRNA by SMG-6, which gives rise to a substantial burden of ribosomal stalls. Yet our work does not clarify how the NMD machinery recognizes a prematurely terminating ribosome, communicates with SMG-6, nor how SMG-6 decides where to cleave the mRNA. The cleavage site of SMG-6 is closely aligned with the PTC, and based on this we speculate that the PIN domain is brought to the stop codon after translation termination by the NMD-eliciting ribosome.

It is important to note that while we concluded that 15–18nt Ribo-seq reads can occur secondary to SMG-6 cleavage, we do not know whether SMG-6 cleaves within ribosomes or not. We do not think that SMG-6 cleaves within *elongating* ribosomes, as such cleavages would have yielded a tight alignment of termination site and 15–18nt Ribo-seq reads. We did not observe such an alignment for many genes (Figures 6 and 7). Furthermore, if ribosomes stall at PTCs and await SMG-6, in a *smg-6* mutant we would expect stalled ribosomes (21nt or 28nt Ribo-seq reads) at the would-be cleavage sites. We also did not observe this (Figure 4A). Thus we do not favor a model in which SMG-6 cleaves within elongating ribosomes. However, it is still possible that SMG-6 cleaves within (or around) a ribosome, though we expect that such a ribosome would be in a non-elongating state and/or a state not captured by existing Ribo-seq protocols. Recent work suggests that ribosomes can persist at and downstream of some PTCs (58,59). It is also possible that SMG-6 is simply brought to and cleaves the mRNA after a ribosome departs, as prevailing models currently suggest (1). We expect that future work clarifying the precise mechanism and substrate for the PIN domain will prove illuminative.

## DATA AVAILABILITY

Data is available through SRA via Bioproject number PRJNA819566. A list of all libraries generated in this study are in Supplementary Table S2. The Arribere Lab pipeline is available at github (https://github.com/arriberelab/arriberelab) and scripts for this study are therein (220606_kimModenaSmg6Paper).

## Supplementary Material

gkac681_Supplemental_Files
