# Supplementary material for: SMG-6 mRNA cleavage stalls ribosomes near premature stop codons in vivo
Source: Nucleic Acids Res. 2022 Aug 11;50(15):8852–66. doi: 10.1093/nar/gkac681 (PMC9410879; doi:10.1093/nar/gkac681)
Supplement: gkac681_Supplemental_Files [file gkac681_supplemental_files.zip › 220627_supplement.pdf]

SUPPLEMENTARY MATERIAL

TABLES

Table S1: ***C. elegans*** **Strains**

Table S2: Libraries

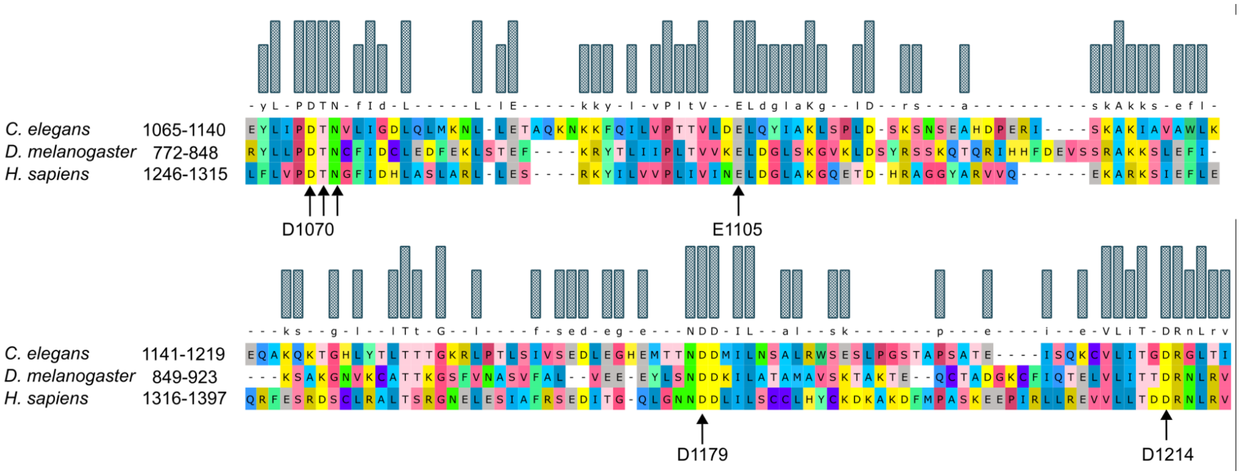

Figure S1. Multiple sequence alignment of SMG-6 PIN domain.
Multiple sequence alignment of the SMG-6 PIN domain between *C. elegans, D. melanogaster,* and *H. sapiens.* Black arrows note residues of interest; all but Asp 1179 were mutated in this study.

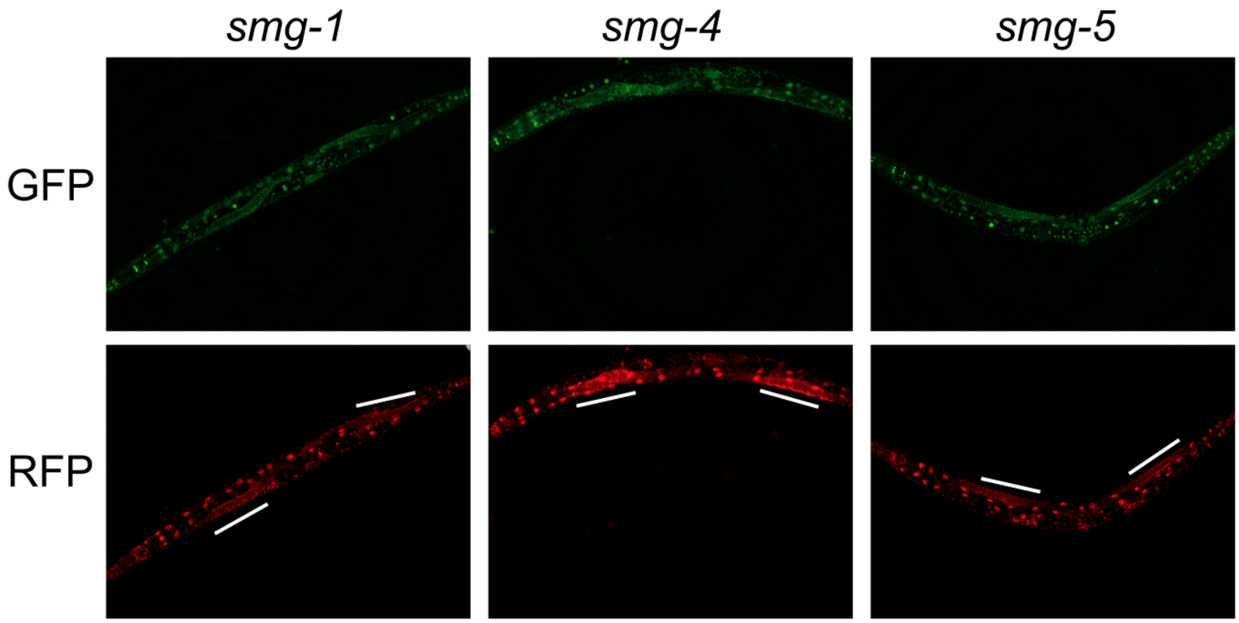

Figure S2. ***smg-1, smg-4,*** **and** ***smg-5*** **are required in both the germline and soma for NMD in** ***C. elegans*****.**
RFP images (top) of *smg-1(e1228)*, *smg-4(az152),* and *smg-5(r860)* with the R2 NMD reporter. White bars indicate the distal arm of the germline.

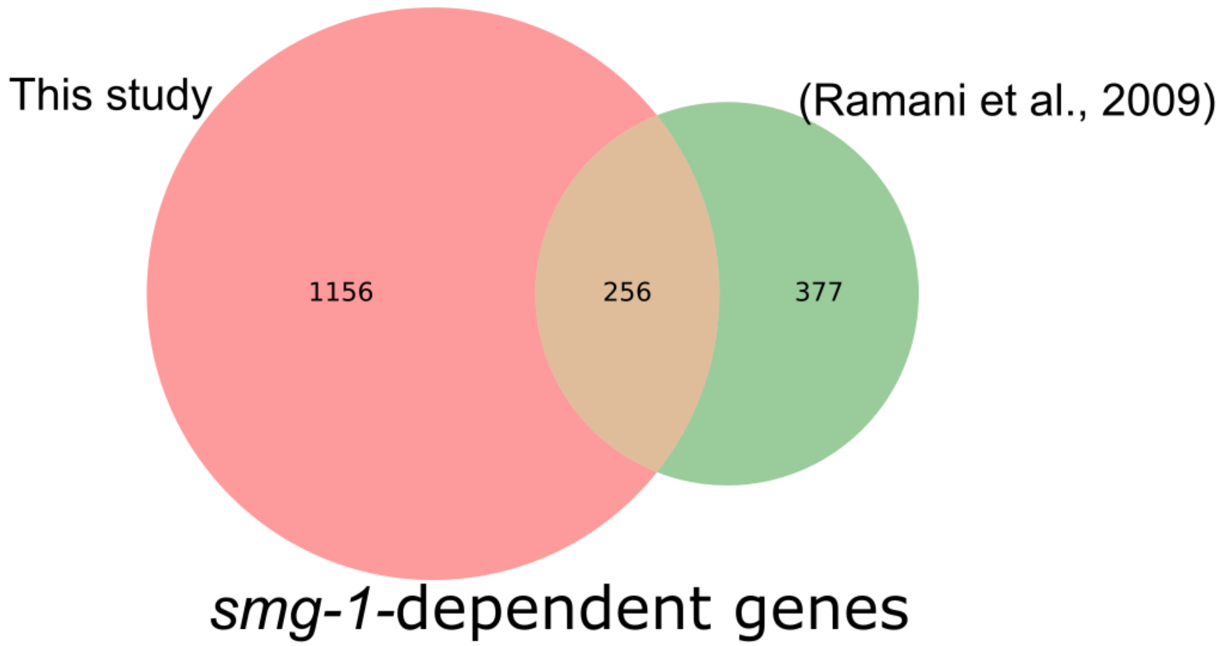

Figure S3. Overlap of NMD targets identified by *smg-1* loss in *C. elegans.*
Venn diagram showing the overlap of NMD targets called by a loss of *smg-1* between this study and published data (38). Differences between the two studies may arise due to differences in animal staging and the methods (RNA-seq vs. microarrays).

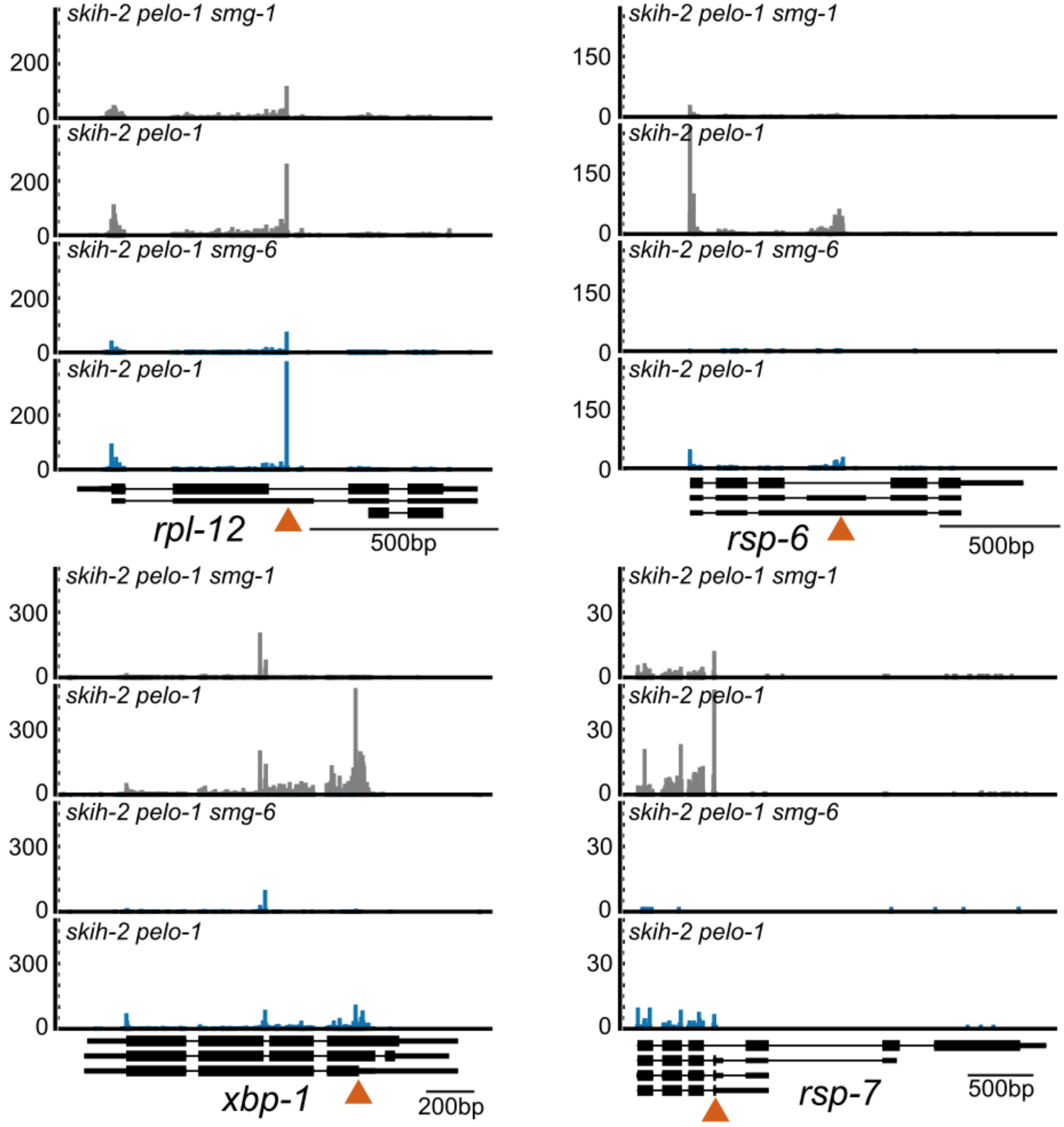

Figure S4. SMG-6 PIN domain is required for the generation of 15-18nt Ribo-seq reads at known NMD targets.

RPM normalized 15-18nt Ribo-seq footprint libraries in *skih-2 pelo-1*, *skih-2 pelo-1 smg-1,* or *skih-2 pelo-1 smg-6(D1070A)* libraries. Orange arrowhead indicates the position of an early stop codon. Scale for each gene on the bottom right. Grey libraries from (10), blue libraries from this study.

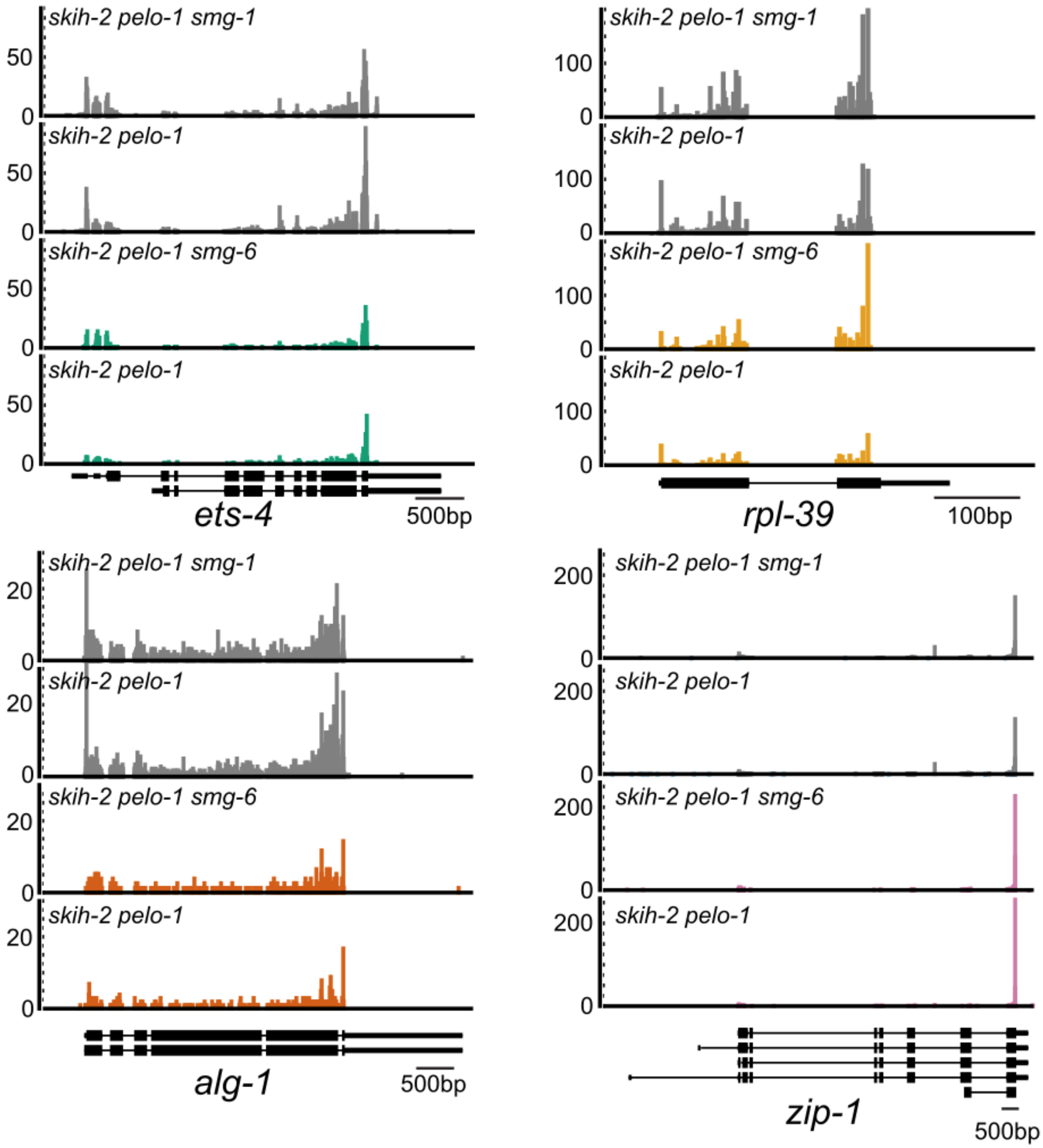

Figure S5. NMD-independent enrichment of 15-18nt Ribo-seq reads on specific genes. RPM normalized 15-18nt Ribo-seq footprint libraries in *skih-2 pelo-1*, *skih-2 pelo-1 smg-1,* and *skih-2 pelo-1 smg-6(D1070A)* libraries. Grey libraries from (10). Colored libraries from this study, and gene-specific coloring matches genes highlighted in Figure 4B. Scale for each gene on the bottom right.

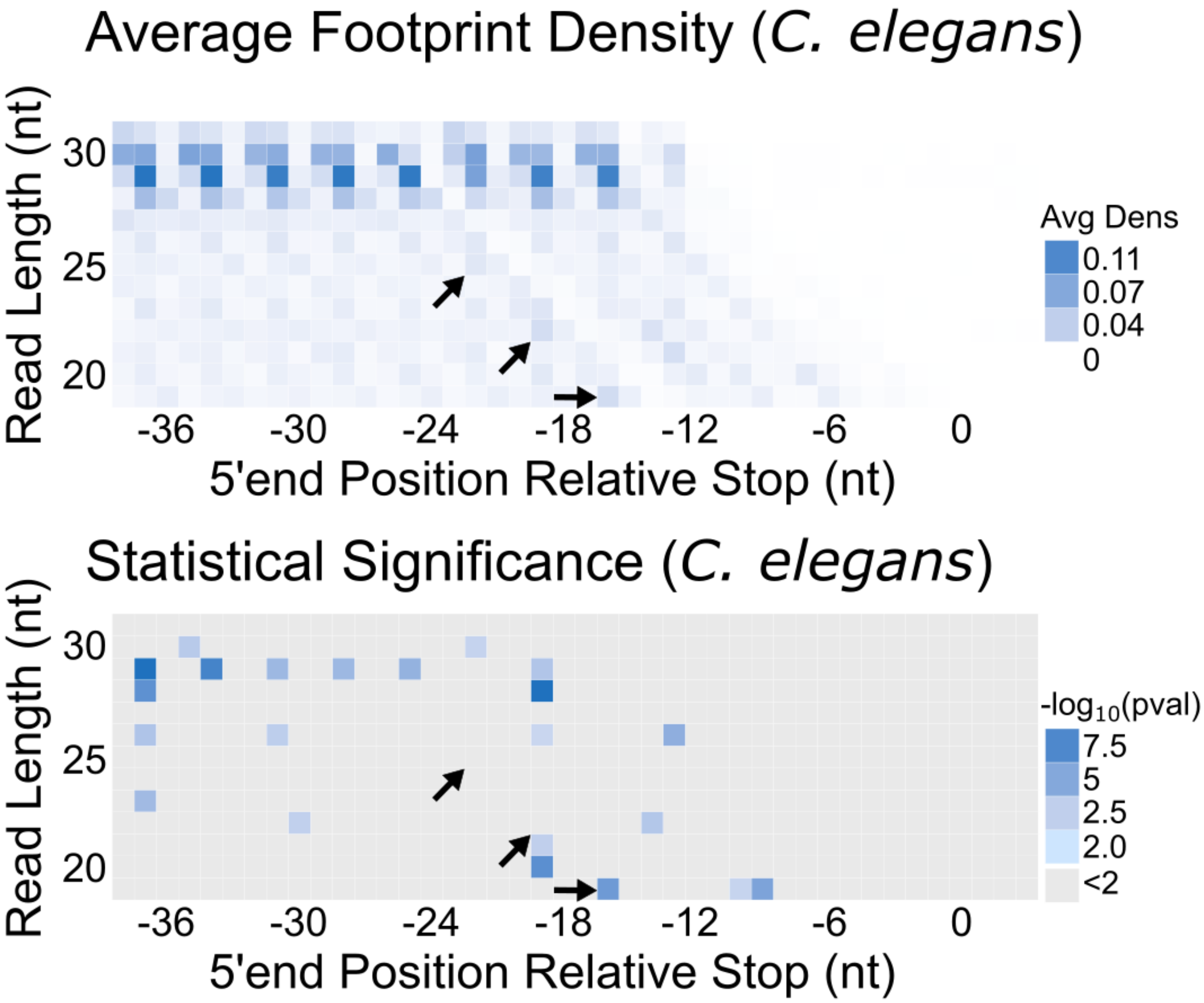

Figure S6. Heatmap of Ribo-seq 5′ ends.
Heatmap showing density of 5′ ends across a range of Ribo-seq footprint sizes relative to stop codons in wild-type *C. elegans* using Ribo-seq libraries made in our hands. Black arrows indicate the expected sites for ribosomes translating to the end of a cleaved message.

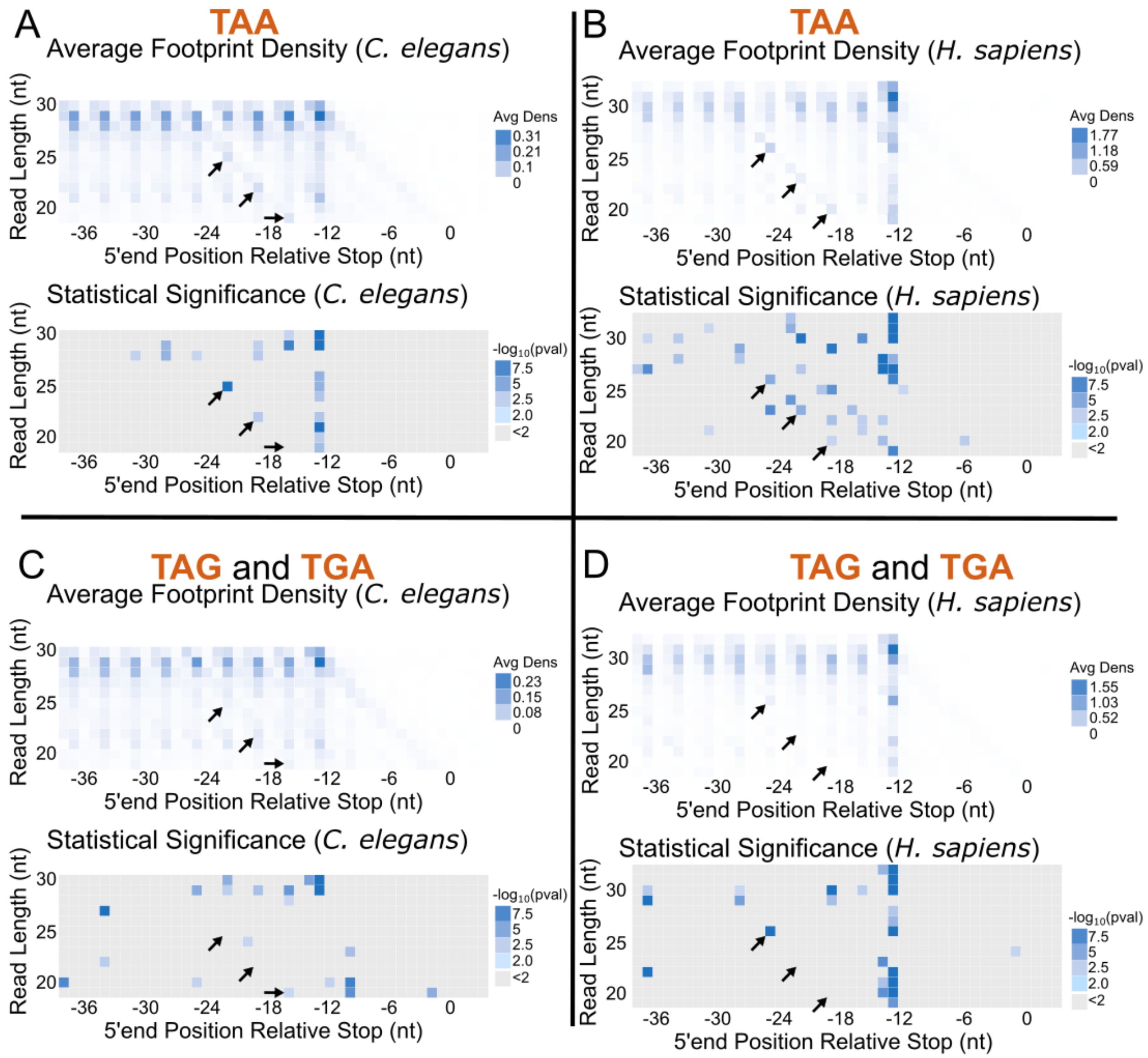

Figure S7. Heatmap of Ribo-seq reads split by stop codon identity.
Heatmap of Ribo-seq reads around the stop codon, as per Figure 5, using published libraries for *C. elegans* (44) and humans (51). Genes were binned into TAA or TAG/TGA according to the nucleotide sequence of their stop codon. We observed similar results when splitting the TAG/TGA group into their respective codons, though doing so exhibited noise due to the relatively few number of TAG- and TGA-ending genes (TAA is the most frequently used stop codon).

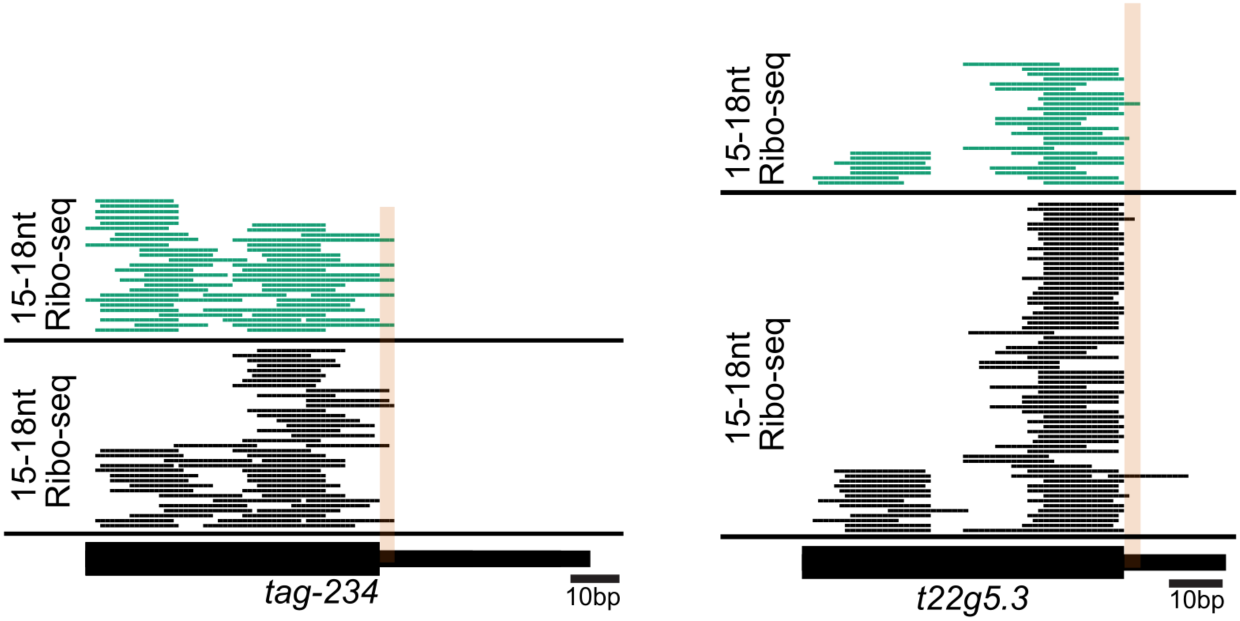

Figure S8. Gene-specific 15-18nt Ribo-seq patterns are reproducible. Short Ribo-seq footprints plotted for two separate genes. Green and black coloring shows biological replicates of 15-18nt Ribo-seq in the *skih-2 pelo-1* background. Orange highlight indicates the location of the stop codon.

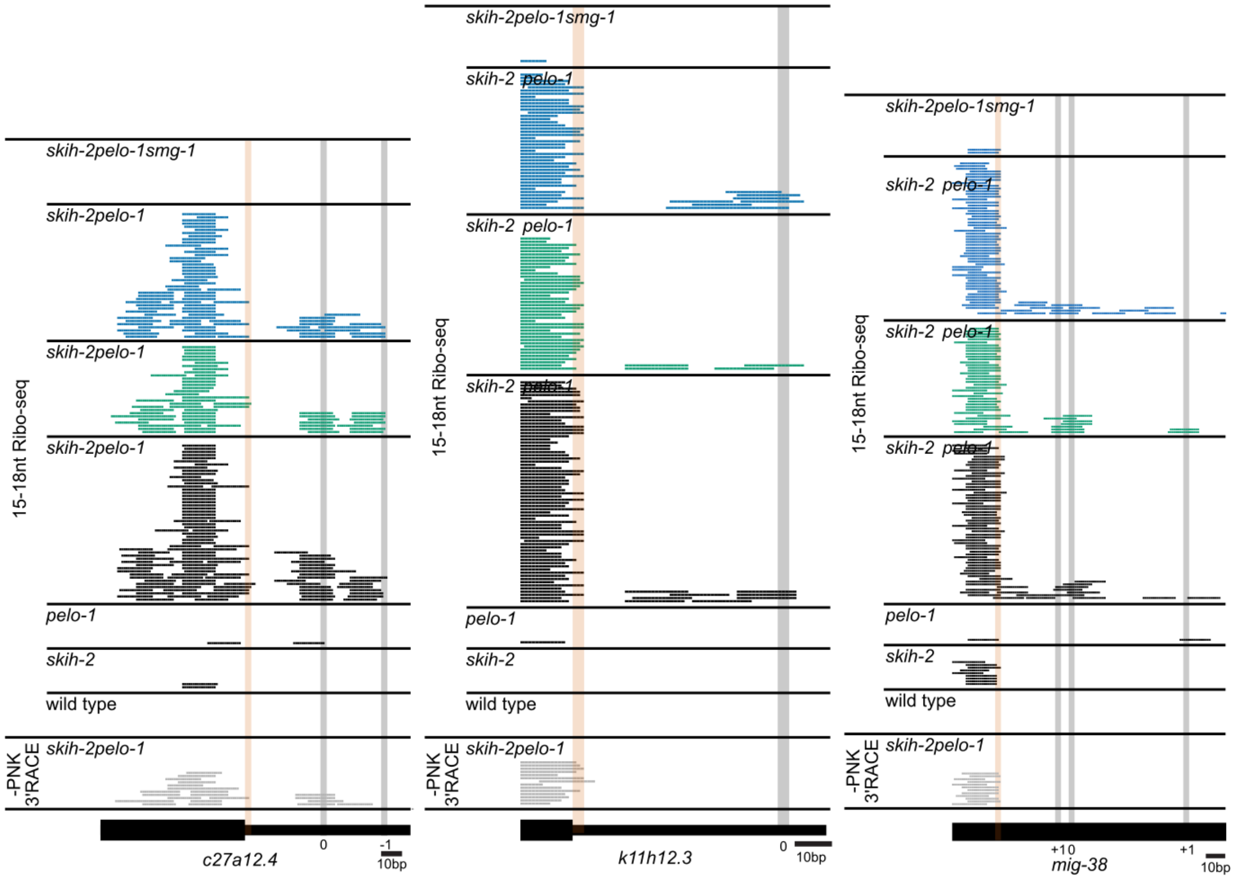

Figure S9. 15-18nt Ribo-seq reads align with 3′ RACE reads. 15-18nt Ribo-seq reads plotted for three separate genes alongside 3′ RACE sequencing. Reads of the same color are of biological samples harvested and libraries prepared in parallel. Orange highlight indicates the location of the stop codon.

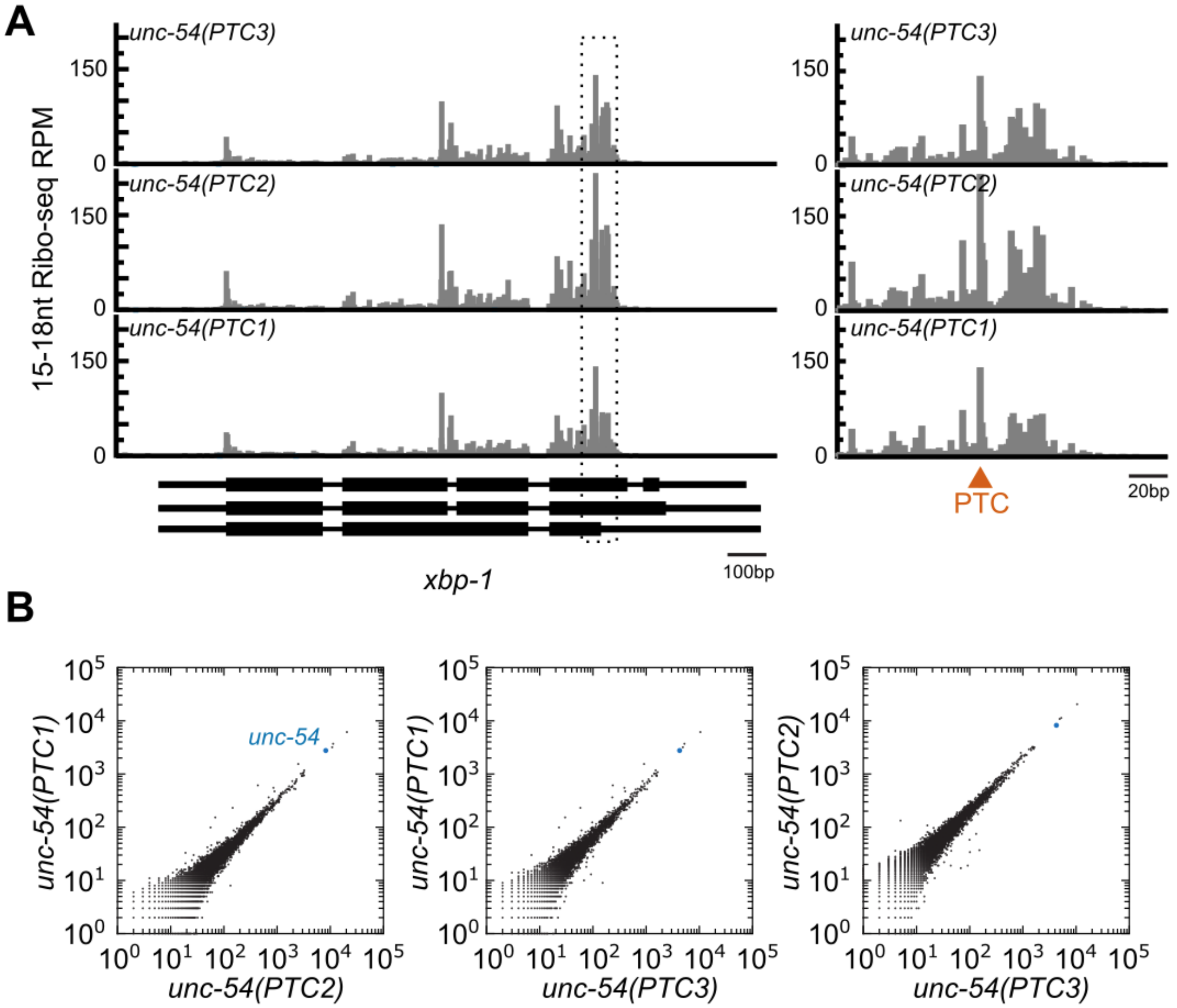

Figure S10. Read density across the *unc-54(PTC)* reporter series is largely similar. A) Read density at *xbp-1* in the different *unc-54* PTC reporter backgrounds (left). Dotted line box notates the area zoomed into (right). Orange arrowhead indicates the location of the PTC. B) Genome-wide read counts across the different *unc-54* PTC reporter libraries. *unc-54* is highlighted in blue. The handful of off-diagonal genes are attributable to the recovery of highly-abundant artifactual reads (rRNA, tRNA fragments) that happen to overlap with a protein-coding gene.

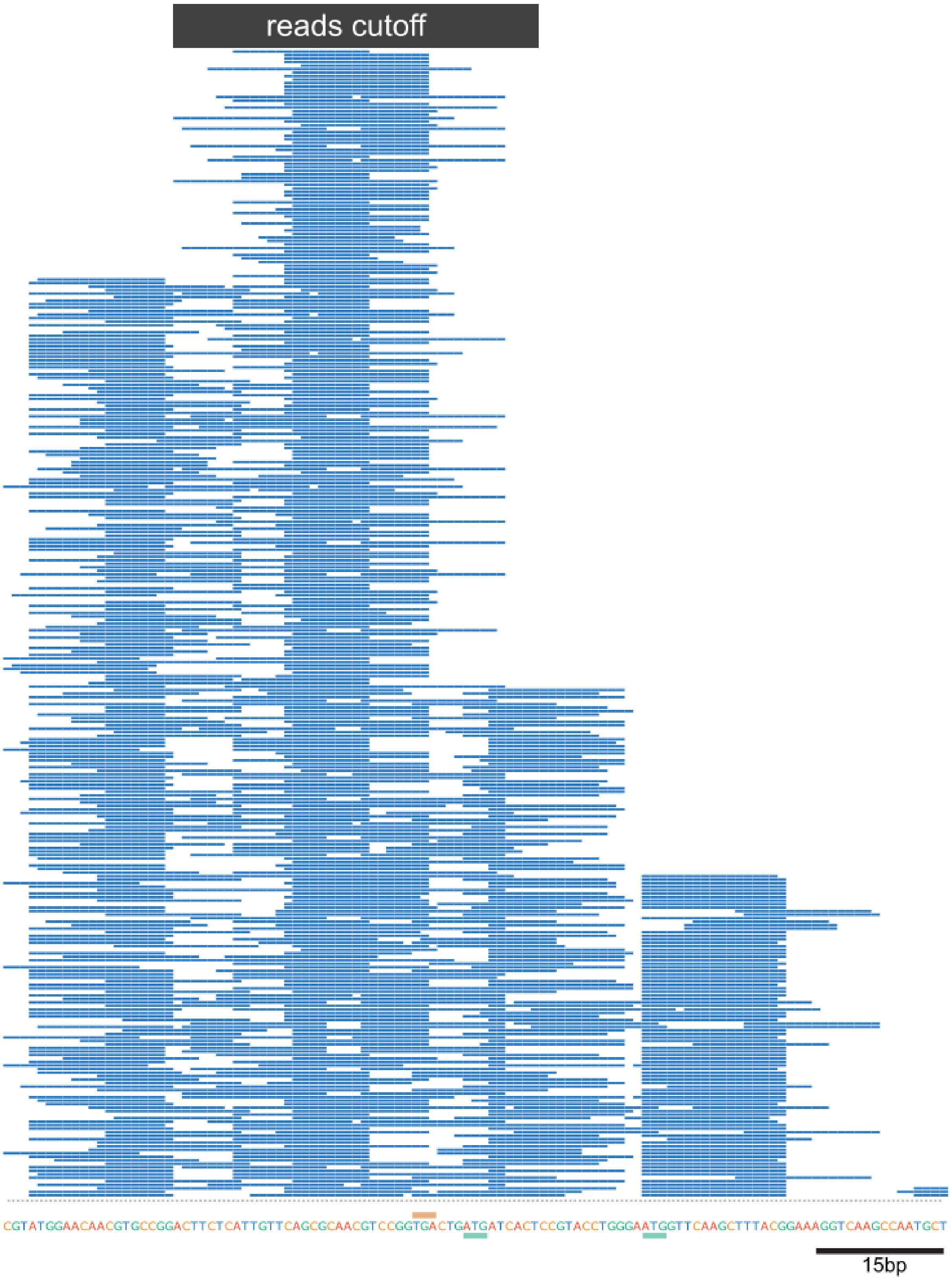
reads cutoff
CGTATGGAACAACGTGCCGGACTTCTCATTGTTCAGCGCAACGTCCGGTGACTGATGATCACTCCGTACCTGGGAATGGTTCAAGCTTTACGGAAAGGTCAAGCCAATGCT
15bp

Figure S11. 15-18nt Ribo-seq reads downstream of the PTC on the *unc-54(PTC2)* reporter. A zoomed view to highlight reads downstream of the in frame stop codon on the *unc-54*(PTC2) reporter. The TGA stop codon is highlighted with an orange bar. Potential start codons (ATG) are indicated with green bars; note both are in-frame with the TGA stop codon.
